# Resveratrol Exerts Dosage and Duration Dependent Effect on Human Mesenchymal Stem Cell Development

**DOI:** 10.1371/journal.pone.0037162

**Published:** 2012-05-16

**Authors:** Lindsay Peltz, Jessica Gomez, Maribel Marquez, Frances Alencastro, Negar Atashpanjeh, Tara Quang, Thuy Bach, Yuanxiang Zhao

**Affiliations:** Biological Sciences Department, California State Polytechnic University at Pomona, Pomona, California, United States of America; University of Udine, Italy

## Abstract

Studies in the past have illuminated the potential benefit of resveratrol as an anticancer (pro-apoptosis) and life-extending (pro-survival) compound. However, these two different effects were observed at different concentration ranges. Studies of resveratrol in a wide range of concentrations on the same cell type are lacking, which is necessary to comprehend its diverse and sometimes contradictory cellular effects. In this study, we examined the effects of resveratrol on cell self-renewal and differentiation of human mesenchymal stem cells (hMSCs), a type of adult stem cells that reside in a number of tissues, at concentrations ranging from 0.1 to 10 µM after both short- and long-term exposure. Our results reveal that at 0.1 µM, resveratrol promotes cell self-renewal by inhibiting cellular senescence, whereas at 5 µM or above, resveratrol inhibits cell self-renewal by increasing senescence rate, cell doubling time and S-phase cell cycle arrest. At 1 µM, its effect on cell self-renewal is minimal but after long-term exposure it exerts an inhibitory effect, accompanied with increased senescence rate. At all concentrations, resveratrol promotes osteogenic differentiation in a dosage dependent manner, which is offset by its inhibitory effect on cell self-renewal at high concentrations. On the contrary, resveratrol suppresses adipogenic differentiation during short-term exposure but promotes this process after long-term exposure. Our study implicates that resveratrol is the most beneficial to stem cell development at 0.1 µM and caution should be taken in applying resveratrol as an anticancer therapeutic agent or nutraceutical supplement due to its dosage dependent effect on hMSCs.

## Introduction

Resveratrol (3,5,4′ – hydroxystilbene) is a naturally occurring product found in a number of plants, including grapevine, peanut, blueberry, cranberry, eucalyptus, spruce and the Itadori plant (*Polygonum cuspidatum*) [1], [2], [3], [4]. Numerous *in vitro* and animal studies have shown that resveratrol possesses a wide range of biological effects that could benefit human health, including anticancer chemopreventive and chemotherapeutic activity [5], [6], [7], [8], [9], [10], [11], [12], [13], [14], [15], [16], [17], [18], [19], [20], [21], [22], [23], antioxidation [24], [25], [26], prolonged lifespan from the yeast to short-lived vertebrates [27], [28], [29], [30], [31], protection from neurotoxicity, ischemia and neuron degeneration [32], [33], [34], [35], [36], [37], [38], [39], protection from cardiovascular diseases or injury [40], [41], [42], [43], [44], offsetting the effects of high-calorie diet [45], promoting osteogenesis [10], [11], [46], [47], [48], [49] and inhibiting adipogensis [48], [50], [51], [52]. The *in vitro* and *ex-vivo* anticancer activity of resveratrol on various human cancer cell types was achieved by inhibiting free radical formation [24], inducing cell cycle arrest and apoptosis [6], [7], [15], [16], [17], [18], [19], [53], [54], [55], [56], or suppressing the STAT3 signaling pathway [13]. More recently resveratrol was also shown to inhibit self-renewal and induce apoptosis in human cancer stem cells [12], [57], [58]. On the other hand, the *in vivo* effect of resveratrol in prolonging lifespan was largely attributed to the stimulation of Sirtuins, which deacetylate and destabilize the activity of p53, resulting in delayed apoptosis and prolonged cell survival [27], [59], [60].

Interestingly, there appears to be a dichotomy between the anticancer (pro-death) and pro-survival effect presented by resveratrol that has yet to be fully explained. Such functional discrepancies appear to have resulted from different concentrations of resveratrol exposed to different cells in different experimental settings. For example, the majority of the *in vitro* and *ex vivo* studies used resveratrol at 10 µM or higher concentrations, at which it demonstrated anti-proliferation and pro-apoptotic activity [6], [7], [8], [9], [10], [12], [13], [15], [16], [17], [18], [24], [55], [56], [57]. However, except for the study with *saccharomyces cerevisiae* in which resveratrol was applied at 10 µM in culture medium and found to increase life span by 70% [27], other studies eliciting the lifespan-extending effect of resveratrol were all carried out *in vivo* using animal models. The animals were given resveratrol orally in various regimens without being assessed of the bioavailability of resveratrol and/or its metabolites [28], [29], [30]. While absorption of resveratrol through oral ingestion is high, its bioavailability has been demonstrated to be low in humans. An oral dose of 25 mg resulted in a peak plasma level of 2 µM and a half-life of around 9.2 h [61]. Similarly low bioavailability has been observed in mice, with plasma concentration of resveratrol detected at 1.3 µM in mice fed with an oral dose of 100 mg/kg/day [17]. Such low *in vivo* bioavailability suggests that its observed lifespan-extending effect was derived at a much lower concentration range as compared to that of its anti-proliferation and pro-apoptotic effect. Indeed, differential effects of resveratrol at low vs. high dose/concentrations have been observed in a number of studies [14], [19], [21], [54], [62], [63], [64], [65], [66], [67], [68], though in many cases, low concentration effects were not addressed and focus was placed on the anti-proliferation and pro-apoptosis effect observed at high concentrations instead [14], [19], [21], [62], [63], [64]. In general, resveratrol promotes cell proliferation and inhibits apoptosis at concentrations below 10 µM in tumor cells lines [14], [19], [21], [62], [63], [65], [66], [67], while in normal cell lines such as endothelial cells, growth stimuli were observed at 1 µM or below [54], [64], [68]. Indeed, in one *in vivo* study it was shown that, in contrary to its widely demonstrated anticancer activity *in vitro*, oral uptake of resveratrol at 16.5 mg/kg three times a week by mice xenografted with human breast cancer cells stimulated tumor growth compared to vehicle-treated animals [69], presumably due to low bioavailability *in vivo*.

Despite the lack of full understanding of resveratrol's dynamic actions, studies of this compound have inspired its clinical trials and commercialization as a nutraceutical [70], [71]. One potential cell target for resveratrol through oral or intravenous injection is the adult stem cells residing in different tissues and organs. However, limited studies have examined the effect of resveratrol on stem cell development [10], [11], [46], [47], [72], [73]. Moreover, only one study used normal primary human stem cells, which briefly described the enhancing effect of resveratrol in a dosage dependent manner (0.01 µM to 10 µM) on the proliferation rate and osteogenic differentiation of hMSCs after short-term exposure [11]. HMSCs reside in a number of tissues or organs, including bone marrow, adipose tissue, umbilical cord blood and fetal liver, and are one of the most abundant adult stem cell types in the body. They are capable of self-renewal and upon receiving appropriate external stimuli, could differentiate into multiple mature cell types, such as osteocytes, adipocytes and chondrocytes. As with other adult stem cell types, they are thought to serve as the reservoir for maintaining tissue homeostasis and tissue repair *in vivo*.

To gain better understanding of the effect of resveratrol on hMSCs development (adipose tissue derived), we examined how short-term (≤2 weeks) vs. long-term (4–10 weeks) exposure to resveratrol at various concentrations (0.1, 1, 5 & 10 µM) could alter these cells' capacity to self-renewal and differentiate. Our results demonstrated a dosage dependent effect of resveratrol on hMSCs self-renewal as a result of its combinatorial effect on cell senescence rate, cell doubling time and cell proliferation rate, as well as a dosage- and, in the case with adipogenic differentiation, time-dependent effect on hMSCs differentiation.

## Results

### Resveratrol exerts dosage dependent enhancing vs. inhibitory effect on the self-renewal rate of hMSCs

**Figure 1:**
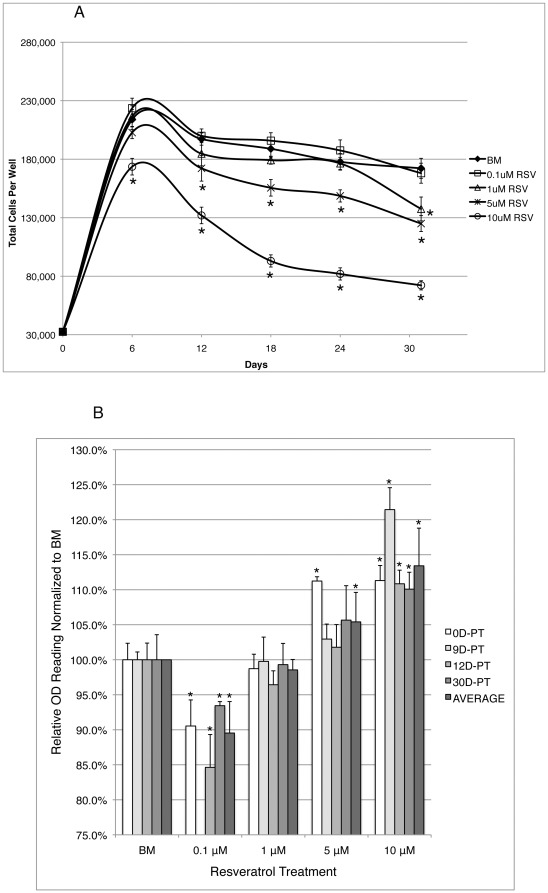
Resveratrol exerts dosage dependent enhancing vs. inhibitory effect on the self-renewal rate of hMSCs. **A**). Cells were plated at equal density and cultured in different concentrations of resveratrol continuously for 36 days during which cells were counted and split at equal ratio every 6 days. *: p<0.01. **B**). Cells pretreated with resveratrol or BM for 0 (0D-PT), 9 (9D-PT), 12 (12D-PT) or 30 (30D-PT) days were seeded at 8000 cells/well and continued to culture in corresponding media until resazurin assay. Error bars represent standard deviation (triplicates in each treatment condition). *: p<0.05 vs. BM.

During short-term culture of hMSCs, it was visually noted that cells treated with 5 or 10 µM resveratrol expand slower than cells treated with α-MEM basal media (BM) solvent or lower concentration resveratrol. To confirm this observation, cells at passage 4 (P4) were plated at equal density and cultured with resveratrol or BM (controlled for the same amount of BM in 10 µM resveratrol) for 30 days. Every 6 days cells were manually counted and split identically among all treatment groups. Figure 1.A. shows that while 0.1 µM resveratrol seemed to provide slight growth advantage over BM treated cells, higher concentrations showed a dosage dependent inhibitory effect. To further confirm and examine whether the observed enhancing/inhibitory effect occurred only during early treatment or throughout the duration, cells pretreated for 0 (0D-PT), 9 (9D-PT), 12 (12D-PT) or 30 (30D-PT) days of resveratrol or BM were plated at equal low density and treatment was continued until cells of one treatment group reached 100% confluency (regardless of the confluency of the other groups), when resazurin assay was carried out to assess the relative number of live cells in each treatment condition. The OD reading of resazurin solution is inversely correlated to the numbers of live cells [74]. At all 4 different pretreatment time points, 0.1 µM resveratrol treatment resulted in the lowest OD reading and therefore the highest cell numbers, whereas higher concentrations of resveratrol resulted in a dosage dependent increase in OD readings (Figure 1.B.), demonstrating a similar trend observed by manual cell counting. The results indicated that resveratrol enhanced cell self-renewal rate at 0.1 µM, had no significant effect at 1 µM, and inhibited cell self-renewal at 5 and 10 µM.

Cell self-renewal rate could be affected by cytotoxicity, cell senescence rate, cell apoptosis rate, cell cycle progression rate (cell doubling time) and cell proliferation rate (percentage of cells entering cell cycle). To examine which are the contributing factors to the altered cell self-renewal rates in response to resveratrol treatments, the following assays were carried out.

### Resveratrol treatment does not cause acute cytotoxicity in hMSCs

**Figure 2:**
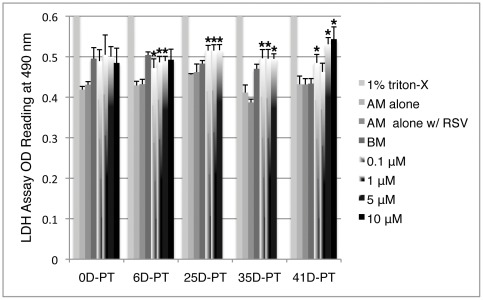
Resveratrol does not cause acute cytotoxicity in hMSCs at concentrations tested. Untreated P4 cells (0D-PT) or cells pretreated for 6 (6D-PT), 25 (25D-PT), 35 (35D-PT) or 41 days (41D-PT) were plated at equal density and then subjected to 24 hours of treatment in corresponding BM or resveratrol media before LDH assay. AM: assay media; AM alone: no cells; AM alone w/RSV: no cells and the highest concentration of RSV in the group was used; 1% triton X-100 in AM: positive control (all values >2.5). Data presented were the mean values of each treatment type. Error bars represent standard deviation. *: p<0.05 vs. BM.

To examine whether resveratrol at high concentrations could be toxic to the cells, a lactate dehydrogenase (LDH)-based cytotoxicity assay was carried out, which measures LDH activity released from the cytosol of damaged cells into the supernatant. Untreated P4 cells (0D-PT) were plated at equal density across all wells on 96-well plates and then subjected to BM or resveratrol treatment, with 12 wells per condition. After 24 hours, LDH activity was measured and no significant difference was observed among BM and resveratrol treatment groups (Figure 2). Similar results were obtained when LDH activity was subsequently measured in combined supernatant from 5 days of daily collection for each treatment group with fresh media change after each collection (data not shown). This indicates that resveratrol does not cause acute cytotoxicity at concentrations tested. In addition, LDH activity was examined in cells pretreated for 6 (6D-PT), 25 (25D-PT), 35 (35D-PT) or 41 days (41D-PT) of BM or resveratrol and plated at equal density across different treatment groups (Figure 2). In 6D-PT cells, resveratrol treatment at 5 µM or below had reduced LDH activity as compared to BM, whereas at 10 µM it is not significantly different from BM or the other resveratrol treatment groups. In the other pretreatment groups, all resveratrol treatments slightly but significantly increased LDH activity compared to the BM group (except for 1 µM at 41D-PT), but significant differences between low (0.1 and 1 µM) vs. high (5 or 10 µM) resveratrol treatment only appeared in 41D-PT cells (P<0.01). This indicated that any potential acute cytotoxicity by resveratrol was unlikely a pivotal factor in conferring its differential effect on cell self-renewal rate at different concentrations, as it consistently promoted cell self-renewal at 0.1 µM and exerted opposite effect at 5 and 10 µM throughout both short- and long-term treatments as shown above.

### Resveratrol treatment does not exert significant apoptotic effect in hMSCs

**Figure 3:**
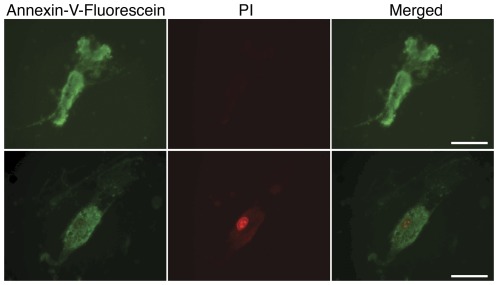
Apoptosis assay of hMSCs. Representative images of cells stained positive for annexin-V-fluorescein (green) alone or positive for both annexin-V-fluorescein and propidium iodide (PI, red) are presented. Scale: 50 µm.

To examine its potential effect on cell apoptosis rate, untreated cells (0D-PT) or cells pretreated with BM or resveratrol for 30 days (30D-PT), were subjected to 6 or 5 days of treatment respectively after equal density plating, followed by co-staining with Annexin-V-Fluorescein and Propidium iodide (PI) solution. Annexin-V (green) labels both apoptotic and necrotic cells whereas PI (red) labels only necrotic cells (Figure 3). In 0D-PT-6D-RSV cells, no clear trend could be identified among all treatment groups in either the percentages of apoptotic (G) or necrotic (G+R) cells (Table 1). In 30D-PT-5D-RSV cells, all resveratrol treatments (except for 1 µM) resulted in higher percentages of necrotic (G+R) cells than the BM group (Table 1). In addition, there appeared to be a very subtle dosage dependent upward trend in the percentages of apoptotic (G) cells among the resveratrol treatment groups (Table 1).

In the acute cytotoxicity assay described earlier, LDH activity was measured on cells treated with BM or resveratrol in AM media for 24 hrs after equal density plating. Such time restriction is necessary in order to minimize the effect of cell density change on the LDH readout after prolonged cell culture. To examine whether apoptotic rate is affected by resveratrol treatment under similar conditions, cells pretreated with BM or resveratrol for 25 days were plated at equal density and subjected to only 24 hours of culture in corresponding regular CM-based treatment media (25D-PT-1D-RSV) before co-staining with Annexin-V/PI solution. The percentages of necrotic (G+R) cells, but not apoptotic (G) cells, trended higher in all resveratrol treatment groups than the BM group. In addition, no trend was observed across resveratrol treatments (Table 1). Since the LDH assay required using an assay media (AM) with low fetal bovine serum (1%), instead of the regular CM media, for reconstituting BM or resveratrol treatment solution, we also carried out Annexin V/PI staining on 35D-PT cells plated at equal density followed by 24 hours of BM or resveratrol treatment in AM media as in the LDH assay (35D-PT-1D-RSV). Similar to the 25D-PT-1D-RSV cells, all resveratrol treatments (except for 1 µM) resulted in higher percentages of necrotic (G+R), but not apoptotic (G) cells than the BM group, and no trend could be identified among the resveratrol treatment groups in either category (Table 1). In contrary to in regular CM media, it was noted that in AM media, majority of green cells were also stained red. The increased percentages of necrotic cells, but not apoptotic cells, could have contributed to the observed slight increase of LDH activities by resveratrol-treated vs. BM-treated groups in the LDH assays carried out on the 25D-PT and 35D-PT cells.

Overall, the above results indicated that resveratrol had no significant effect on cell apoptosis rate at concentrations tested during short-term treatment and therefore would not have likely contributed to its dosage-dependent effect on cell self-renewal rate, but cells subjected to prolonged resveratrol exposure may demonstrate a subtle dosage dependent increase in apoptotic rate, which could contribute to the long-term effect of resveratrol on cell self-renewal.

### Resveratrol exerts dosage dependent pro-survival vs. pro-senescence effect on hMSCs

**Figure 4:**
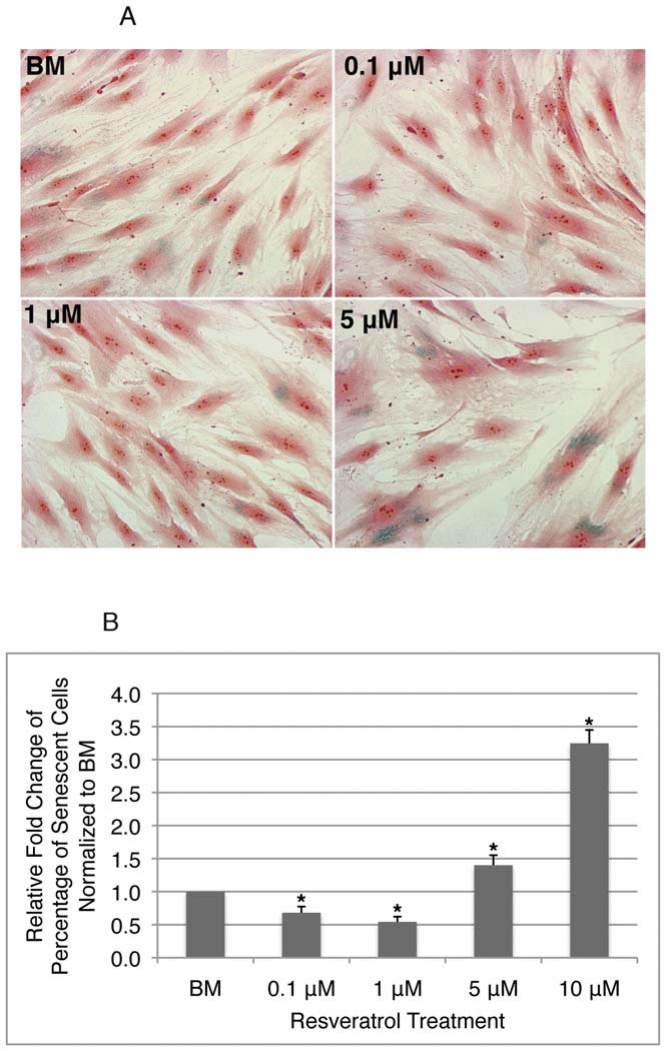
Resveratrol exerts dosage dependent anti- vs. pro-senescence effect on hMSCs. **A**). Cells were stained in X-gal (blue color) and neutral red solution (red color). Images were taken at 200× magnification. **B**). Percentages of senescent cells vs. total cells were determined based on images taken. At least 184 total cells from each treatment condition were counted. Column represents the relative fold changes of percentage of cells undergoing senescence in each treatment group normalized to the value obtained in the BM treated cells. Data was obtained from three independent experiments. Error bars represent standard deviation. *: p≤0.01 vs. BM.

To examine cell senescence rate, beta-galactosidase activity was measured as an indicator of cellular senescence [75]. Early passage cells (P4 & P5, corresponding to 0D-PT and 6D-PT cells respectively) were plated at similar density before being subjected to resveratrol or BM treatment until they reach about 90% or less confluency. Cells were then fixed and stained in X-gal solution and counterstained in neutral red solution (Figure 4.A). Compared to BM treated cells, cells treated with 0.1 or 1 µM resveratrol had significantly less cells stained positive for beta-galactosidase activity compared to those treated with 5 or 10 µM resveratrol (Figure 4.B.), indicating that at 0.1 or 1 µM, resveratrol inhibits senescence, but at 5 and 10 µM, it promotes senescence. To examine whether cells pretreated with resveratrol or BM for extensive period of time respond similarly, cells pretreated with resveratrol or BM for 30 days (30D-PT) were plated at similar density and continued in their corresponding media until senescence assay. At 0.1 µM, resveartrol remained slightly inhibitory to senescence compared to BM, while at 1 µM or higher, it drastically increased the senescence rate at 3–9 times higher than BM in a dosage dependent manner (Figure S1). The results indicated that resveratrol consistently inhibited senescence at 0.1 µM and promoted senescence at 5 or 10 µM, while at 1 µM, it inhibited senescence during short-term pretreatment but significantly enhanced senescence after long-term exposure.

**Table 1:** Resveratrol does not affect cell apoptosis rate in hMSCs.

	0D-PT-6D-RSV		30D-PT-5D-RSV	
	AVG (G+R)	AVG(G)	AVG (G+R)	AVG(G)
BM	0.49%	2.46%	1.05%	6.29%
0.1 µM	0.44%	3.43%	1.32%	5.87%
1 µM	0.27%	2.04%	1.06%	6.80%
5 µM	0.51%	2.81%	1.54%	7.07%
10 µM	0.17%	1.69%	n/a	n/a
	25D-PT-1D-RSV		35D-PT-1D-RSV	
	AVG (G+R)	AVG(G)	AVG (G+R)	AVG(G)
BM	0.31%	7.58%	3.64%	1.04%
0.1 µM	0.62%	4.75%	5.04%	1.11%
1 µM	0.48%	4.34%	3.61%	0.88%
5 µM	0.67%	3.92%	5.55%	0.89%

Annexin-V-FLUO staining was carried in cells pretreated with BM or resveratrol for 0 or 30 days, followed by 6 (0D-PT-6D-RSV) or 5 (30D-PT-5D-RSV) more days of treatment respectively after equal density plating, and cells pretreated with BM or resveratrol for 25 or 35 days, followed by 1 day of additional treatment after equal density plating (25D-PT-1D-RSV or 35D-PT-1D-RSV). The percentage of apoptotic cells stained green (G) alone or necrotic cells stained both green and red (G+R) were counted from two wells of each treatment group. The total cells counted from 2 wells of each treatment group exceeded 4000 cells.

### Long-term resveratrol pretreatment prolongs cell-doubling time in hMSCs in a dosage dependent manner

**Figure 5:**
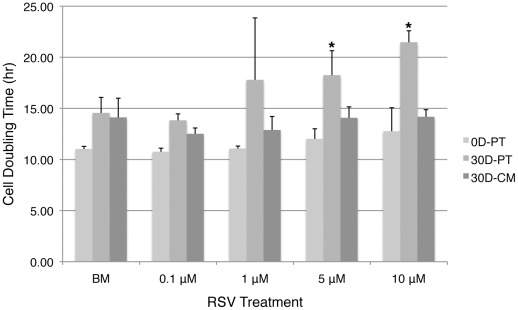
Resveratrol pretreatment prolongs cell-doubling time of hMSCs in a dosage dependent manner. Untreated early passage cells (0D-PT), untreated late passage cells (30D-CM) and cells pretreated with resveratrol or BM for 30 days (30D-PT) were plated at 1–2 cells/well density and exposed to the same resveratrol or BM treatment for 72 hours or longer for cell-doubling time assay. Data presented are the mean values of 2 independent experiments for each experimental set. Error bars represent standard deviation. *: p<0.05 vs. BM.

To examine cell-doubling time, both untreated early passage cells (0D-PT) and cells pretreated for 30D with BM or RSV (30D-PT) were plated at 1–2 cells/well density in CM in 96-well plates and changed to BM or RSV the next day (0 hr time point). Total of 10 wells were selected for each treatment group and cells in each well were recorded at 0 hr and every 12-hr intervals until 72 hrs or longer. To minimize error, cell doubling time at each time point beyond 0 hr was calculated based on the average cell counts from all 10 wells for each treatment group and the average doubling time was then calculated from all time points. Media was changed every 48 hrs. For 0D-PT cells, the cell-doubling time appeared to be steadily reduced by 0.1 µM resveratrol and increased by 5 or 10 µM resveratrol as compared to BM, though none of the differences was considered as statistically significant (P>0.05) (Figure 5). On the other hand, for 30D-PT cells, cell-doubling time was clearly significantly increased in 5 µM or 10 µM treated cells compared to BM treated cells. To verify whether this increase in cell-doubling time was a result of accumulating effect of resveratrol treatment over time or a result of increased sensitivity of response to resveratrol in aging cells, age-matched cells that have been cultured in CM (30D-CM) were subjected to identical assay. Similar to the 0D-PT cells, no statistically significant differences were observed between resveratrol vs. BM treated groups, though the overall cell-doubling time of the 30D-CM group was increased as compared to that of the 0D-PT group (P<0.01) (Figure 5), which is consistent with visual observations that late passage cells take longer to reach 100% confluency than earlier ones when plated at the same density (data not shown). The above results indicated that long-term resveratrol exposure at 5 or 10 µM significantly prolonged cell-doubling time.

### Resveratrol increased cell proliferation rate in a time and dosage dependent manner

**Figure 6:**
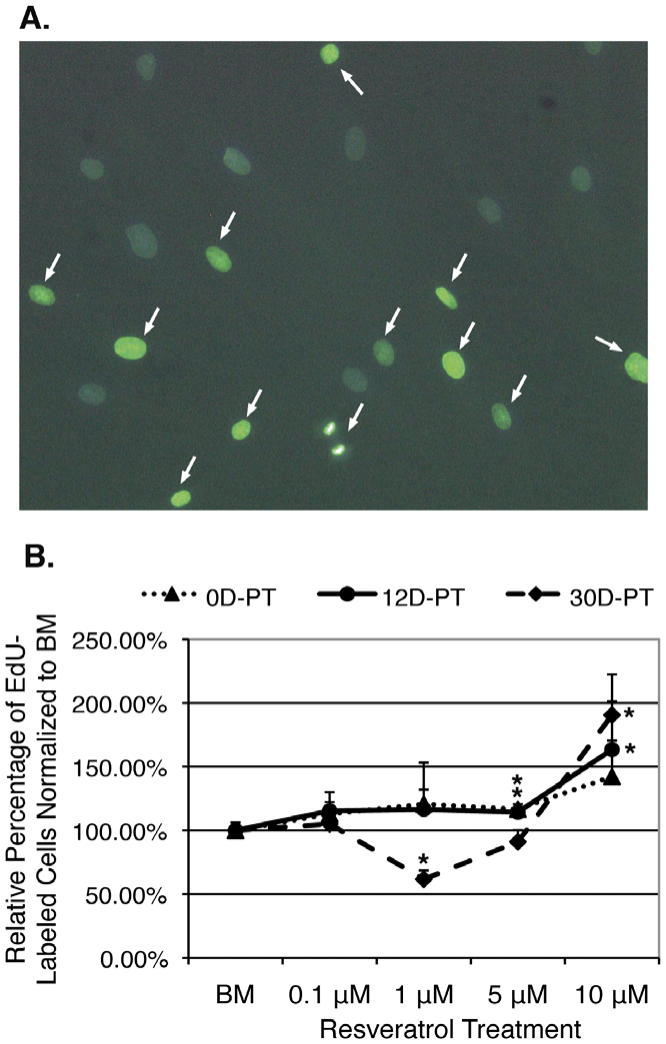
Resveratrol pretreatment affects cell proliferation rate of hMSCs in both time and dosage dependent manner. **A**). Duplicate wells of cells pretreated with resveratrol or BM for 0 (0D-PT), 12 (12D-PT) or 30 (30D-PT) days, followed by 6 (0D-PT) or 5 (12D-PT & 30D-PT) more days of treatment respectively after similar density plating were fixed and stained at 6 or 12 hrs post EdU addition. Cells labeled with EdU were green (indicated by arrows) whereas unlabeled cells appeared grey from Hoescht 33342 staining (image taken using UV/Blue dual filter at 200× magnification). **B**). Percentages of green cells were normalized to the value in the BM treated cells. Error bars represent standard deviation. *: p<0.05 vs. BM.

To examine cell proliferation rate, cells pretreated in resveratrol or BM for 0 (0D-PT), 12 (12D-PT) or 30 (30D-PT) days were subjected to Click-iT EdU (5-ethynyl-2′-deoxyuridine) assay. Cells were plated in similar density in their perspective media and cultured for 6 (0D-PT) or 5 (12D-PT & 30D-PT) more days respectively until reaching around 50–70% confluency when EdU, a nucleoside analog of thymidine, was added. Duplicate cells were fixed 6 hours or 12 hours later for detection of incorporated EdU. The labeling duration was determined based on the average cell-doubling time for hMSCs and two different time points were chosen for parallel confirmation. The percentage of green (EdU-labeled proliferating cells) vs. grey (Hoechst-labeled total cells) cells was determined by counting cells across each well of 24-well plates (in between 800 to 2100 cells/well, depending on treatment condition) (Figure 6A & B). The results demonstrated that during short period of resveratrol treatment (6–17 days), there was a dosage dependent enhancing effect on the proliferation rate of hMSCs, culminating at 10 µM. However, after prolonged treatment (35 days), proliferation rate dropped significantly in response to 1 µM resveratrol, but remained up regulated by 10 µM.

### Resveratrol increased the percentage of S-phase cells in a dosage dependent manner

**Figure 7:**
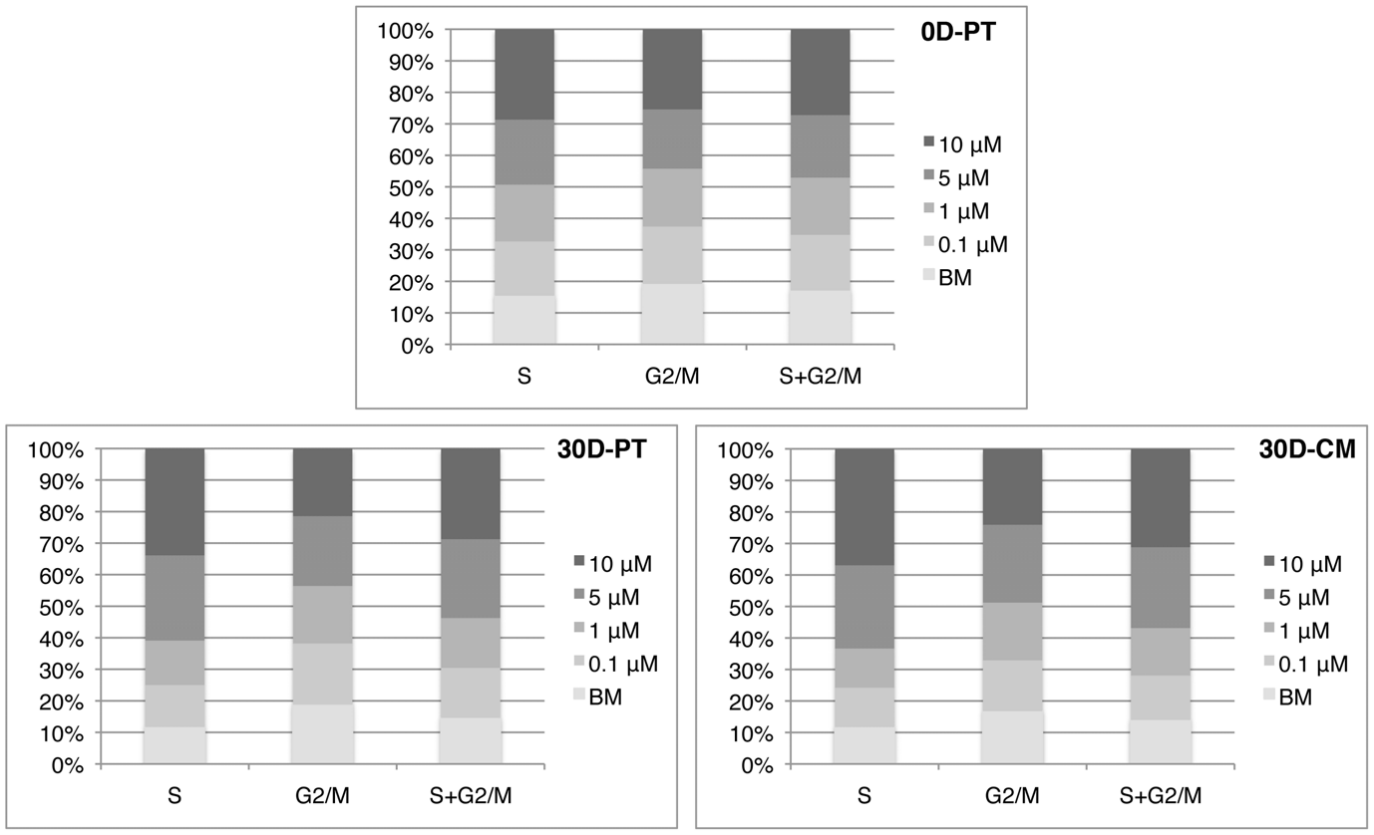
Resveratrol increased the percentage of S-phase cells in a dosage dependent manner. Flow cytometry was performed to analyze cell cycle distributions on cells pre-exposed to resveratrol or BM for 0 (0D-PT) or 30 (30D-PT) days, followed by 6 (0D-PT) or 4 (30D-PT) more days of treatment respectively after equal density plating, and cells cultured in regular hMSCs media (CM) for 30 days prior to 4 days of resveratrol or BM treatment following equal density plating (30D-CM). Stacked columns represent the relative distribution of cells in S, G2/M or (S+G2/M) phases in each treatment group normalized to the value in the BM treated cells (see Table S1). Data presented are the mean values of 2 or 3 independent experiments for each experimental set. All duplicate experiments had similar outcomes.

Increased cell-doubling time and cell proliferation rate would suggest increased representation of S and/or G2/M phase cells in high concentration resveratrol treated cells. To verify this, we performed flow cytometry based cell cycle analysis on cells pretreated with resveratrol or BM for 0 (0D-PT) or 30 (30D-PT) days, followed by 6 (0D-PT) or 4 (30D-PT) more days of treatment respectively after equal density plating. As shown in table S1 and figure 7, in both cases, resveratrol caused a dosage dependent increase in the representation of S phase cells. In 0D-PT cells, only 10 µM resveratrol resulted in increased representation of G2/M phase cells, whereas in 30D-PT cells, both 5 and 10 µM did. The discrepancy observed between short- and long- term resveratrol treatment could have been due to changes in cellular response as a result of long-term resveratrol treatment or as a result of cellular aging *in vitro* for the long-term treated cells. To distinguish these two scenarios, cells were cultured in regular CM for hMSCs in parallel to cells cultured in resveratrol or BM for 30 days, followed by 4 days of resveratrol or BM exposure before cell cycle analysis (30D-CM). The distribution of G2 phase cells were increased by both 5 and 10 µM resveratrol treatment, suggesting that it was *in vitro* cellular aging that had caused the discrepancy observed between short- vs. long- term exposure. Additional repeat with cells pretreated with BM or resveratrol for 42 days (42D-PT) followed by 4 additional days of culture after equal density plating demonstrated similar results as those of 34D-RSV (Table S1). The above results indicate that regardless of age or duration of pre-exposure to resveratrol, the percentage of hMSCs residing in both S and G2/M phases (S+G2/M) is significantly increased by resveratrol in a dosage dependent manner, consistent with its effect in cell doubling time and cell proliferation rate.

In summary of the above results, at low concentration (0.1 µM), resveratrol promotes cell self-renewal of hMSCs by reducing cell senescence rate and perhaps by increasing cell proliferation rate and reducing cell-doubling time as well, whereas at high concentrations (5 or 10 µM), resveratrol inhibits cell self-renewal of hMSCs by significantly increasing cell senescence rate and cell-doubling time, which might be partly offset by increased cell proliferation rate. Such inhibitory effect may also be exacerbated by enhanced apoptotic rate after long-term exposure.

### Resveratrol regulates the expression of genes implicated in cell cycle regulation, cell senescence and longevity

**Figure 8:**
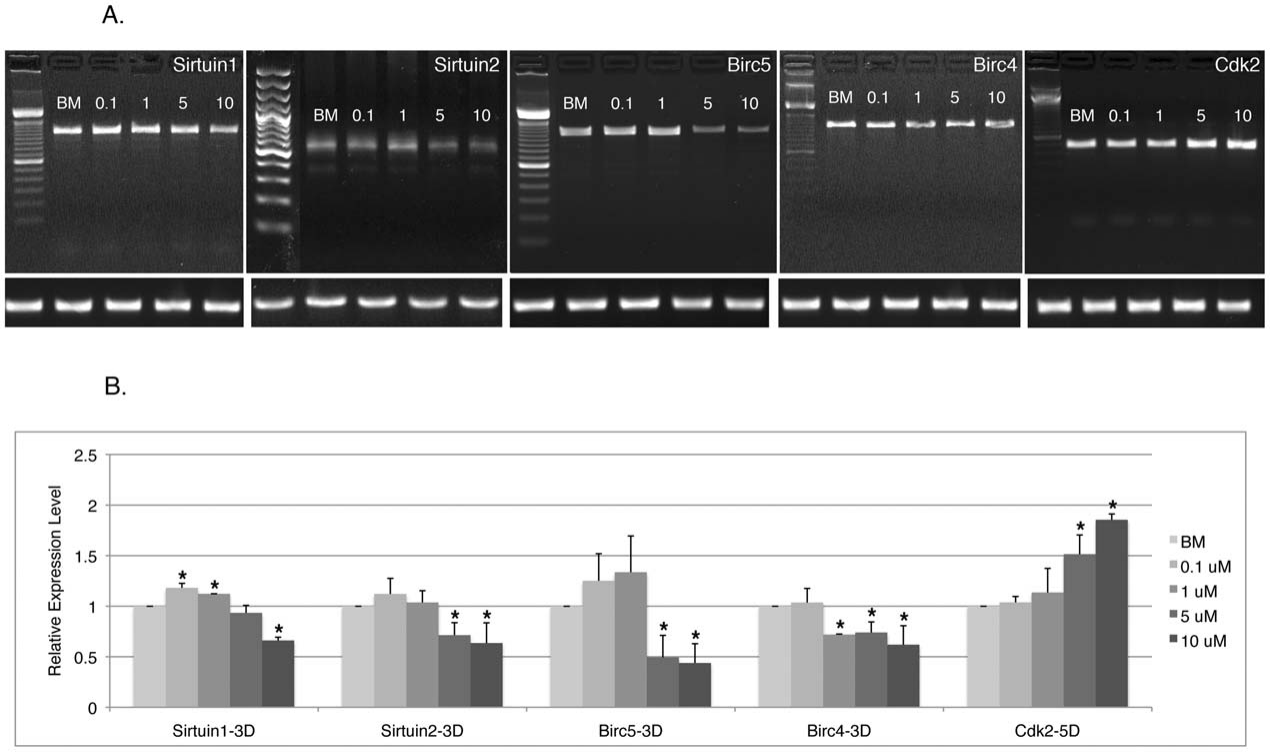
Resveratrol regulates the expression of genes implicated in cell cycle, cell senescence and longevity regulation. **A**). Representative gel images of gene expression examined by semi-quantitative RT-PCR on cells pretreated with BM or resveratrol for 3 or 5 days. Expression of internal control gene *Hsp90* from the same batch of *cDNA* for each gene is shown in the bottom row. **B**). Expression of each gene in resveratrol treated cells was quantified relative to that in BM treated cells and normalized by the expression level of housekeeping gene *Hsp90*. Data shown are the mean values of three repeats. Error bars represent standard deviation. *: p<0.05 vs. BM.

In light of the observed effect of resveratrol on cell cycle, senescence and proliferation, we examined the expression of a number of genes implicated in those biological processes, including *c-Myc*, *Cdk2*, *p16*, *p21*, *p53*, *Cyclin D1*, *Birc4*, *Birc5*, *Sirtuin1* and *Sirtuin2*, in cells pretreated with resveratrol for 3 days (3D) or 5 days (5D). Sirtuin1 and Sirtuin2 are thought to be critical players in prolonging life span [27], [59], [60]. Birc4/XIAP and Birc5/Survivin are members of the Inhibitors of Apoptosis (IAPs) family that suppress caspase activities [76]. Cdk2 promotes cells entering cell cycle whereas p21 is a cell cycle inhibitor. Among those genes, *Sirtuin1*, *Sirtuin2*, *Birc4*, *Birc5* and *Cdk2* consistently demonstrated altered expression in response to resveratrol. *Sirtuin1*, *Sirtuin2*, *Birc4* and *Birc5* were weakened in 5 or 10 µM resveratrol pretreated cells as compared to BM treated cells, whereas *Cdk2* demonstrated enhanced expression (Figure 8). The relative changes observed in all genes appeared subtle but statistically significant, and correlate to increased senescence rate and higher percentage of S phase cells in response to high concentration resveratrol exposure.

### Resveratrol promotes osteogenic differentiation of hMSCs in a dosage dependent manner

**Figure 9:**
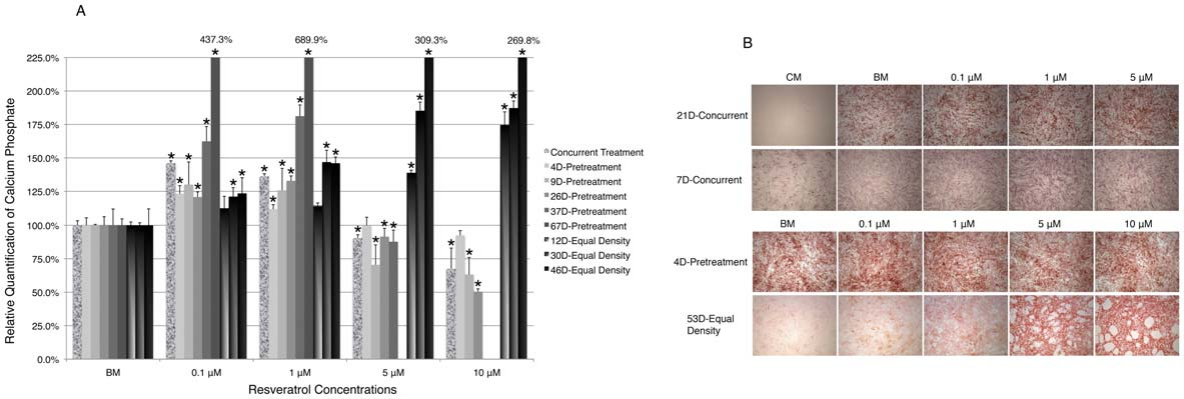
Resveratrol modulates osteogenic differentiation of hMSCs in a dosage dependent manner. **A**). Calcium phosphate deposit was stained by alizarin red solution and subsequently quantified under three different treatment schemes: concurrent treatment, pretreatment and equal density. Missing columns are a result of detached cells. **B**). Images of alizarin red stained cells under different treatment conditions. 7D-Concurrent treatment cells were stained for alkaline phosphatase activity. Concurrent treatment: cells were exposed to both resveratrol and OIM throughout the differentiation duration; Pretreatment: cells were cultured in BM/resveratrol conditioned media continuously for certain days prior to OIM induction; Equal density: Cells were cultured in BM/resveratrol conditioned media continuously for certain days and re-plated at equal density before OIM induction. Except for the pretreatment groups, all experiments were repeated 3 times independently, with triplicates in each experimental set. A representative data set is presented for each group and data shown are the relative mean values of triplicates normalized to the value of the BM control cells in each group. Error bars represent standard deviation. *: p<0.05 vs. BM.

HMSCs can be induced to undergo osteogenic or adipogenic differentiation *in vitro* when exposed to appropriate stimuli. We therefore assessed how different concentrations of resvertrol could affect both processes after short- or long-term exposure. When exposed to osteogenic inducing media (OIM), hMSCs differentiate into mature bone cells characterized by extracellular matrix mineralization [77]. We began by examining how concurrent treatment of resveratrol with OIM could affect the outcome. Early passage cells (P4) at 100% confluency were exposed to OIM and resveratrol at different concentrations for 14 to 21 days before alizarin red staining, which detects calcium phosphate deposit, a major component of mineralized extracellular matrix. Consistently, resveratrol enhanced osteogenic differentiation at 0.1 or 1 µM as indicated by increased calcium phosphate deposit, but exerted opposite effect at 5 or 10 µM (Figure 9A & B). This is also confirmed by examining the activity of Alkaline Phosphatase (ALP), an early marker of osteogenic differentiation [78], [79]. ALP staining was clearly enhanced by both 0.1 and 1 µM resveratrol, with increased intensity in individual cells and increased cell density (Figure 9B). In the presence of 5 µM resveratrol however, ALP staining intensity is enhanced in some individual cells as well, but the overall intensity is reduced due to reduced cell density (Figure 9B).

To further gauge the thresholds at which resveratrol exerts enhancing or inhibitory effect under such condition, cells were treated with OIM and resveratrol in a broader concentration range including 0.01, 0.1, 1, 2, 3 and 5 µM. At 0.01 µM, resveratrol had minimum effect on OIM activity. At 0.1 µM it reached maximum enhancing effect, which gradually decreased as concentration increased (Figure S2). In addition, we examined the effect of resveratrol on hMSCs derived from bone marrow (hMSCs-Bm), which are characteristically identical to hMSCs derived from adipose tissue (hMSCs-Ad), the cell type chosen in this study. Not surprisingly, these cells responded to resveratrol similarly as hMSCs-Ad (Figure S2).

To examine how resveratrol treatment alone could change the cells' capacity to undergo osteogenesis, cells were pretreated with BM or resveratrol for 4, 9, 26, 37 and 67 days (4D, 9D, 26D, 37D or 67D respectively), with identical passaging among all treatment groups throughout the culture, followed by OIM induction. Similar to concurrent treatment, resveratrol promoted osteogenesis at 0.1 or 1 µM, but inhibited this process at 5 or 10 µM (Figure 9A & B).

However, since resveratrol enhanced cell self-renewal rate at 0.1 µM and inhibited cell self-renewal at 5 and 10 µM, we examined whether cell numbers could have played a role in the above outcome by re-plating resveratrol-pretreated cells at equal density among all treatment groups before OIM induction in order to compensate the effect of resveratrol on cell self-renewal. Interestingly, under such condition, resveratrol clearly exerted a dosage dependent enhancing effect at all concentrations after long-term pretreatment (30 or 46 days, Figure 9A & B), suggesting that resveratrol intrinsically promoted osteogenic differentiation capacity of hMSCs in a dosage dependent manner, though at high concentrations such effect was offset by its inhibitory effect on cell self-renewal.

### Resveratrol exerts differential effect on adipogenic differentiation of hMSCs in a time dependent manner

**Figure 10:**
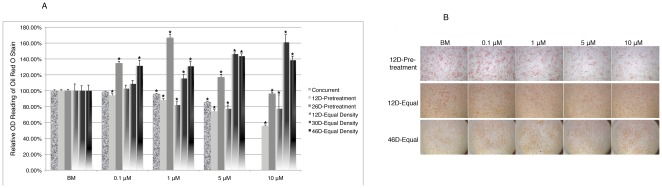
Resveratrol modulates adipogenic differentiation of hMSCs in a dosage dependent manner. **A**). Oil droplets were stained by Oil-Red-O solution and subsequently quantified under three different treatment schemes: concurrent treatment, pretreatment and equal density. **B**). Images of Oil-Red-O stained cells under different treatment conditions. Concurrent treatment: cells were exposed to both resveratrol and AIM throughout the differentiation duration; Pretreatment: cells were cultured in BM/resveratrol conditioned media continuously for certain days prior to AIM induction; Equal density: Cells were cultured in BM/resveratrol conditioned media continuously for certain days and re-plated at equal density before AIM induction. Except for the pretreatment groups, all experiments were repeated 3 times independently, with triplicates in each experimental set. A representative data set is presented for each group and data shown are the relative mean values of triplicates normalized to the mean value of the BM control cells in each group. Error bars represent standard deviation. *: p<0.05 vs. BM. Images were taken at 8× except for bottom row in B) (6.3×).

When exposed to adipogenic inducing media (AIM), hMSCs differentiate into mature fat cells characterized by oil droplets inside the cells. Similar to our study with osteogenic differentiation, the effect of resveratrol on adipogenic differentiation was carried out in three different schemes: concurrent treatment, pretreatment and equal density plating. When cells were exposed to AIM and resveratrol/BM concurrently for 18 days, there was a dosage dependent inhibitory effect (Figure 10A), which was also observed with hMSCs-BM (data not shown). When cells were pretreated with BM or resveratrol continuously for 12 or 26 days (12D or 26D respectively), with identical passaging among all treatment groups throughout the culture, followed by AIM induction, they exhibited differential outcomes. After 12D pretreatment, resveratrol inhibited adipogenesis in a dosage dependent manner similar to concurrent treatment. However, after 26D pretreatment, resveratrol promoted adipogenesis, though the effect was diminished at 10 µM, which was likely due to the counteracting effect of resveratrol on cell self-renewal at this concentration (Figure 10A & B). To compensate its effect on cell self-renewal, cells pretreated with resveratrol or BM for 12, 30 or 46 days (12D, 30D or 46D respectively) were re-plated at equal density before being subjected to AIM induction. Except for at 0.1 µM, resveratrol inhibited adipogenesis after 12D pretreatment but promoted it after 30D pretreatment (Figure 10A & B). This suggests that the enhancing effect of resveratrol at 0.1 µM after 26D-pretreatment without cell density correction was likely due to enhanced cell self-renewal. Interestingly, after 46D pretreatment, resveratrol exhibited enhancing effect on adipogensis at all concentrations after equal density plating (Figure 10A & B). The above results indicated that resveratrol had an overall inhibitory effect on adipogensis during short-term treatment, but after long-term treatment, it reversed to enhance adipogensis.

### Resveratrol regulates the expression of genes implicated in osteogenesis and adipogensis

**Figure 11:**
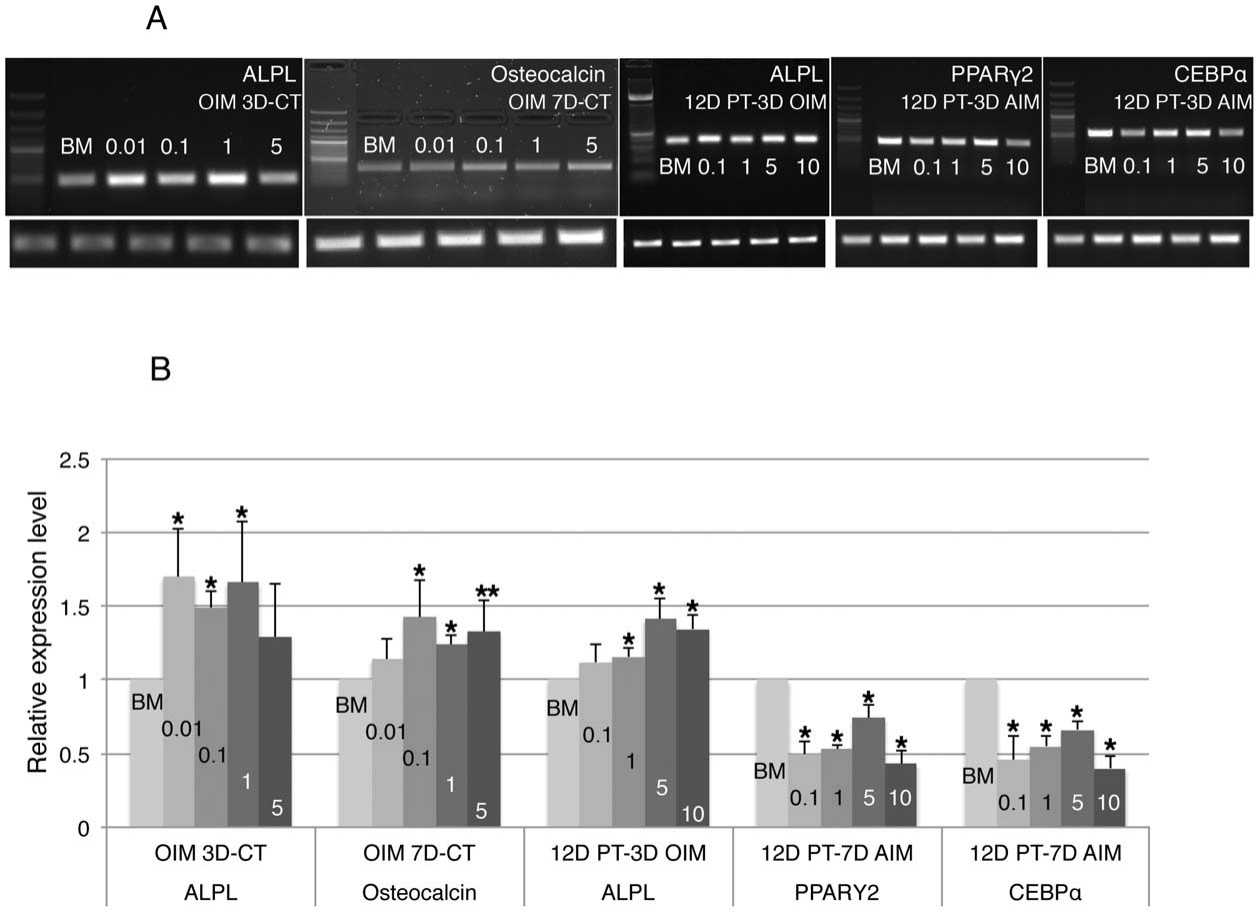
Resveratrol regulates the expression of genes implicated in osteogenesis and adipogenesis. **A**). Representative gel images of gene expression examined by semi-quantitative RT-PCR on cells subjected to concurrent treatment of BM/resveratrol with OIM for 3 days (OIM 3D-CT) or 7 days (OIM 7D-CT), or cells pretreated with BM/resveratrol for 12 days followed by 3 days of OIM (12D PT-3D OIM) or 7 days of AIM (12D PT–7D AIM) exposure. Expression of internal control gene *Hsp90* from the same batch of *cDNA* for each gene is shown in the bottom row. **B**). Expression of each gene in resveratrol treated cells was quantified relative to that in BM treated cells and normalized by the expression of housekeeping gene *Hsp90*. Data shown are the mean values of three repeats. Error bars represent standard deviation. Concentration unit: µM. *: p<0.05 vs. BM. **: p = 0.055 vs. BM.

Osteogenic differentiation is characterized by the expression of a number of genes including *ALPL* (liver/bone/kidney specific isoform of Alkaline Phosphatase), an early marker and *Osteocalcin*, a late-stage differentiation marker [79]. On the other hand, adipogenic differentiation is characterized by the expression of *PPARγ2*, *CEBPα* and others [80]. Semi-quantitative RT-PCR was carried out to examine the expression levels of the above genes on cells subjected to 3 or 7 days of BM or resveratrol treatment with OIM (3/7D-CT OIM) or AIM (3/7D-CT AIM) concurrently, and cells pretreated with BM or resveratrol for 12 days followed by 3 or 7 days of OIM (12D PT-3/7D OIM) or AIM (12D PT–3/7D AIM). Expression of *ALPL* was weakly but significantly up regulated in both 3D-CT OIM and 12D PT-3D OIM resveratrol samples vs. BM control samples (Figure 11A & B). Expression of *Osteocalcin* was also slightly up regulated in 7D-CT OIM resveratrol samples vs. BM samples. On the other hand, expression of both *PPARγ2* & *CEBPα* was significantly down regulated in 12D PT-7D AIM resveratrol samples compared to BM samples (Figure 11A & B), and similar results were observed in 3D-CT AIM samples as well (data not shown). The above results were consistent with the phenotypic observation of resveratrol in promoting osteogenesis and inhibiting adipogenesis during short-term exposure.

In summary, our studies demonstrated: I) hMSCs self-renewal was slightly enhanced by resveratrol at 0.1 µM, unchanged at 1 µM but significantly inhibited at 5 or 10 µM, regardless of the pretreatment duration. Such effect was a result of combined actions on cell senescence, cell cycle progression and cell proliferation, whereas apoptosis did not likely play a role during short-term treatment, but may exert a subtle effect after prolonged exposure to resveratrol. At 0.1 µM, resveratrol inhibited senescence rate, without significantly altering cell doubling time or cell proliferation, regardless of pretreatment duration. At 1 µM, short-term resveratrol pretreatment reduced senescence rate, while long-term treatment resulted in significant increase. Neither short- nor long-term treatment had significant effect on cell doubling time but cell proliferation rate was drastically reduced after long-term treatment. In contrary, at 5 or 10 µM, both short- and long-term resveratrol pretreatment significantly increased cell doubling time, cell senescence rate and the percentage of cells in the (S+G2/M) phase. Interestingly, cell proliferation rate based on EdU assay was also significantly stimulated by 5 and 10 µM resveratrol. II) hMSCs were differentially regulated by resveratrol in terms of its osteogenic and adipogenic differentiation capacity. Regardless of the pretreatment duration, resveratrol enhanced osteogenic differentiation capacity of hMSCs in a dosage dependent manner, with the strongest induction at 10 µM, though this was also offset by its inhibitory effect on cell self-renewal at high concentrations. On the other hand, short-term pretreatment inhibited while long-term pretreatment promoted adipogenic differentiation of these cells. Finally, molecular studies also showed differential effect of resveratrol at different concentrations on the expression of a number of genes involved in cell cycle, cell apoptosis, cell survival, adipogensis and osteogenesis, providing some molecular basis for its differential effect on hMSCs self-renewal and differentiation.

## Discussion

Our study revealed distinct and much more dynamic actions on both cell self-renewal and differentiation of hMSCs conferred by different concentrations of resveratrol, as compared to a previous study [11]. At 0.1 µM, resveratrol appears to offer the most benefit, promoting stem cell self-renewal and osteogenesis over both short- and long-term exposure, inhibiting adipogenesis over short-term exposure, but promoting adipogenesis over long-term exposure. At 5 or 10 µM however, resveratrol inhibits cell self-renewal, but regulates osteogenesis and adipogenesis similarly as at 0.1 µM. Corresponding to the opposing effect of resveratrol at low vs. high concentrations on the self-renewal of hMSCs, genes implicated in cell survival (*Sirtuin 1*, *Sirtuin 2*, *Birc5 & Birc4*) were up-regulated by lower concentration resveratrol, but inhibited by higher concentration resveratrol. It is noteworthy that while hMSCs exposed to high concentration resveratrol exhibited increased cell senescence rate and cell doubling time, their proliferation rate was increased too, which corresponded to enhanced *Cdk2* expression. Increase in proliferation rate however may have limited effect to offset the inhibitory effect on self-renewal conferred by the increases in senescence and cell doubling time, as cells entering cell cycle would end up undergoing S-phase arrest and have prolonged cell-doubling time. The mechanism underlying the biphasic dose response on gene expression regulation is unclear and would be of great interest for future studies. It is important to note that similar type of dose-dependent effect (termed hormesis) has been observed with numerous other compounds, and related studies have been collected in a database [81], [82], [83].

It is intriguing that resveratrol exerts opposing effect on adipogenesis during short- vs. long-term treatment, however it is not clear whether this is due to differential response of young vs. aging cells, as long-term pretreated cells are also older. Our study on cell cycle analysis comparing earlier (0D-PT) vs. later passage cells (30D-CM) demonstrated that there was indeed differential response to the same concentration resveratrol between these two populations (Figure 7 and Table S1). Future studies would be needed to fully elicit potential differential response to resveratrol by young vs. aging cells derived from young vs. old donors respectively, as well as those derived from earlier vs. later passage cells. Along the line, it is important to point out that the effect of resveratrol on hMSCs *in vivo* may also be modulated by estrogen activity present and different individuals (female vs. male; young vs. old) may have different responding thresholds [14]. It is also interesting to point out that the effect of resveratrol on self-renewal has a greater masking effect on the outcome of osteogenic differentiation as compared to on adipogenic differentiation. For osteogenic differentiation, long-term continuous pretreatment with higher concentration resveratrol resulted in significant reduction of osteogenic activity (Figure 9A & B), which was then reversed when cell density was compensated. On the contrary, for adipogenic differentiation, continuous pretreatment with high concentration (5 µM) resveratrol for 26 days still promoted adipogenesis despite its inhibitory effect on cell self-renewal at this concentration, and the enhancing effect was only diminished at 10 µM, which was reversed when cell density was compensated (Figure 10A & B). This is likely due to the fact that cells undergo great clonal expansion during osteogenic differentiation, whereas during adipogenic differentiation, cells undergo very limited clonal expansion ([84] & unpublished data), thus rendering greater inhibitory effect on osteogenic differentiation by high concentrations of resveratrol when density is not compensated.

Our results suggest that to achieve its anticancer effect at 10 µM or higher concentrations, resveratrol might have adverse effect on hMSCs development, and potentially other normal cell types in the body as well. Our results also suggest that people taking resveratrol supplement should take precaution on the dosage intake on a daily basis. Commercial resveratrol is available at between 50 µg and 300 mg per dosage form. A 25-mg oral dose resulted in peak plamsa levels of resveratrol and metabolites at about 2 µM and a plasma half-life of 9.2±0.6 h [61]. While it is not clear whether the metabolites play any significant biological roles, a dosage intake at the lower scale might be more preferable until our understanding of the function of resveratrol and its metabolites is further advanced.

## Materials and Methods

### Cell culture and differentiation

Human mesenchymal stem cells (hMSCs) were purchased from Fisher Scientific (SV3010201) and cultured in Hyclone Advance STEM expansion media (Fisher Scientific, SH30875KT). Passage 4 hMSCs cells were used in all experiments described. For osteogenic differentiation induction, cells were exposed to an osteogenic incuding media (OIM) cocktail composed of 0.05 mM ascorbic acid 2-phosphate (Fisher NC9445523), 10 mM β-glycophosphate (NC9960188) & 0.2 µM dexamethasone (Fisher NC9756434) for 14 to 21 days. For adipogenic differentiation induction, cells were exposed to an adipogenic inducing media (AIM) cocktail composed of 0.45 mM 3-isobutyl-1-methylxanthine (Fisher NC9875083), 10 µM insulin (Sigma I9278-5ML), 1 µM dexamethasone for 18 days.

### Resveratrol solution preparation

Resveratrol 100 µM stock solution was prepared fresh every 6 days by dissolving 2.6 mg of resveratrol powder (Sigma R5010-100 mg) into 100 ml of warm α-MEM (Invitrogen 12571-048) and filtered. Control solvent is α-MEM (BM). Stored at 4°C.

### Resazurin assay

Bioreduction of the dye by viable cells reduces the amount of its oxidized form (blue) and concomitantly increases the amount of its fluorescent intermediate (red). Reduction in blue can be measured spectrophotometrically by monitoring decrease in absorbance at 600 nm (see instructions in Sigma TOX8-1KT). Briefly, 10% resazurin dye solution (diluted with cell culture media) was added to cells and incubated for 2 hours in CO2 incubator. Solution was then transferred to a new empty plate for OD reading at both 600 nm and 690 nm (background).

### Cytotoxicity assay

Cytotoxicity assay was carried out by following instructions in cytotoxicity detection kit (LDH) from Roche (cat # 11644793001). The assay medium (AM) was composed of DMEM (without sodium pyruvate) with 1% FBS. Cells were plated at equal density the day before treatment and LDH enzyme activity was assayed at 24 hours after treatment is initiated. Different treatment groups were plated in identical matching positions with each other, and reaction mixture was added in dim light to half wells of each treatment group sequentially and then to the other half in reversed order in order to minimize any potential effect of differential incubation time as a result of the sequence in adding this reagent. OD reading was derived at 490 nm subtracted by reading at 690 nm using a Biotek ELx800 plate reader and Gen5 ELISA software.

### Apotosis assay

Cell apoptosis assay was carried out by following instructions in Annexin-V-FLUOS staining kit from Roche (cat # 11 858 777 001). Cells stained positive for Annexin-V alone (green) or both Annexin-V and PI (red) from each sample well were counted and imaged using an Olympus IX50 microscope. Cells were then fixed on the same day and stained with nuclear dye DAPI (Invitrogen cat # P36931). Total cell numbers in corresponding sample wells were subsequently counted.

### Cell senescence assay

X-gal solution made of 1 mg/ml X-gal (Sigma B9146-100MG, dissolve in DMSO), 5 mM K3Fe(CN)6 – Potassium ferricyanide (Sigma 7025870), 5 mM K4Fe(CN)6- potassium hexacyanidoferrate(II) (Sigma P9387), 2 mM MgCl2, and 150 mM NaCl in PBS at pH 6.0 is freshly prepared for each use. Cells were washed 3 times with PBS (pH 7.2) after media removal, fixed with 0.5% glutaraldehyde (Sigma G7651) for 5 minutes at RT, washed in PBS (pH 7.2) supplemented with 1 mM MgCl2 (Add 20 µl 1 M MgCl2 into 20 ml of PBS) twice and stained in X-gal solution overnight at 37°C incubator. Stained cells were then rinsed with PBS once, washed with distilled water twice, at 2 minutes each, and counter stained with 0.33% neutral red solution (330 mg/100 ml in PBS) for 3 minutes at RT. Rinsed with PBS. Images were taken using an Olympus IX50 microscope.

### Cell doubling time assay

Cells were prepared in single cell suspension, diluted to 2-cell/80 µl media density and plated into 96-well plates with 80 µl/well cell solution. Wells were examined the next day and 10 wells containing 1–2 cells were chosen for each treatment condition and average cell number per well was determined at 0 hour (n) and at every 12-hour intervals (N). The number of rounds of cell doubling was determined by LOG(N/n)/LOG(2), which, when divided by total hours, gave rise to cell doubling time.

### Cell cycle assay by DNA content

FACS tubes were pre-coated overnight with 2% Bovine Serum Albumin (BSA) in PBS. Cells were trypsinized into single cell suspension, pelleted and washed with ice cold PBS without Ca+2 or Mg+2, collected by centrifugation and re-suspended in cold PBS (1×10^6^ cells in 0.5 ml) by vortexing using the lowest speed. Cell suspension was then slowly added drop wise to an equal volume of cold absolute ethanol. Cells were then stored at −20°C for at least 2–24 hours, collected by centrifuge at 1600 rpm for 10 min and washed with 3 ml cold PBS twice. Re-suspended cells in 500 µl PI/Triton X-100 staining solution prepared fresh (2 mg DNAse free RNAse A (Sigma R4642) and 0.20 ml of 1 mg/ml PI (Sigma P4864) were added to 10 ml of 0.1% (v/v) Triton X-100 (Sigma) in PBS), and incubated in 37°C water bath for 15–30 minutes. Acquired data (Summit v4.3) on flow cytometer (CyAn ADP-9color, Bechman Coulter) within 48 hours.

### Cell proliferation assay

Cell proliferation rate was determined by following the instructions of the Click-iT EdU Alexa Fluor 488 imaging kit (Invitrogen C10337). EdU labeling time was 6 hours or 12 hours.

### Alkaline phosphatase detection, calcium phosphate staining and Oil-Red-O staining

Alkaline phosphatase activity was detected by following the instruction of the Alkaline Phosphatase Staining kit (Sigma, SD86R). Calcium phosphate was stained by 2% alizarin red solution PH 4.1–4.3 (Fisher AC40048-0250). Briefly, cells were fixed in 10% formalin (Fisher SF100-4) for 15 minutes at RT, rinsed with distilled water 3 times, 5 minutes each, and incubated in alizarin red solution for at least 20 minutes, followed by rinsing with water for 4 times, 5 minutes each. Cells were left air dry before imaging and quantification. Quantification was carried out by following instructions in the Osteogenesis Assay kit (Millipore ECM815). Oil droplets inside mature fat cells were stained by Oil-Red-O solution (Fisher NC9773107). Briefly, cells were fixed in 10% formalin for 20 minutes, rinsed with distilled water 3 times, washed in 100% isopropylene glycol for 5 minutes, incubated in Oil-Red-O solution for 30 minutes, washed with 85% isopropylene glycol for 5 minutes and rinsed with distilled water 3 times before imaging and quantification. For quantification, cells were air-dried, extracted with pure isopropyl alcohol (Fisher A426P) and OD was measured at 510 nm.

### Gene expression by reverse transcription (RT)-PCR analysis

Total RNA was isolated from cells with the RNeasy kit (QIAGEN 74104). SUPERSCRIPT II reverse transcriptase (Invitrogen 11752050) was used for RT. Primer sequences and PCR condition for each gene is summarized in table S2.

### Statistical analysis

Unpaired student *t*-test was used to evaluate the statistical differences between two treatment groups.

## Supporting Information

Figure S1 **Resveratrol exerts dosage dependent anti- vs. pro-senescence effect on hMSCs.** Percentages of senescent cells vs. total cells were determined based on images taken on X-gal stained cells pretreated with resveratrol or BM for 30 days followed by 5 more days of treatment after equal density plating. At least 283 total cells from each treatment condition were counted. Column represents the relative amount of cells undergoing senescence in each treatment group normalized to the value of the BM treated cells. Data was obtained from one experiment.
(TIF)

Figure S2 **Resveratrol exerts dosage dependent effect on osteogenic differentiation of hMSCs derived from both the adipose tissue (hMSCs-Ad) and bone marrow (hMSCs-Bm).** Cells were exposed to resveratrol/BM and OIM concurrently for 14 days before calcium phosphate assay. In hMSCs-Ad, resveratrol reached maximum enhancing effect at 0.1 µM, which gradually decreased as its concentration increased. Similar effect was also observed in hMSCs-Bm (concentrations at 2 and 3 µM were not examined). Data shown are the mean values of triplicates. Error bars represent standard deviation. *: p<0.05 vs. BM.
(TIF)

Table S1 **Resveratrol induces S-phase arrest in hMSCs in a dosage dependent manner.** Flow cytometry was performed to analyze cell cycle distributions on cells pre-exposed to resveratrol or BM for 0 (0D-PT) or 30 (30D-PT) days, followed by 6 (0D-PT) or 4 (30D-PT) more days of treatment respectively after equal density plating, and cells cultured in regular hMSCs media (CM) for 30 days prior to 4 days of resveratrol or BM treatment following equal density plating (30D-CM). The percentage of cells in S, G2/M or (S+G2/M) phases in each treatment group was normalized to the value of the BM treated cells. Data presented are the mean value from 2 or 3 independent experiments for each experimental set, except for the 42D-PT set (cells pretreated with resveratrol or BM for 42 days were plated at equal density, followed by 4 more days of treatment before analysis), which was derived from a single experiment. All duplicate experiments had similar outcomes.
(DOC)

Table S2 **Primer sequences and PCR conditions.** This table enlists the primer sequences and PCR conditions used for gene expression examination by RT-PCR.
(DOCX)

## References

[1] Soleas GJ, Diamandis EP, Goldberg DM (1997). Resveratrol: a molecule whose time has come? And gone?. Clin Biochem.

[2] Rimando AM, Kalt W, Magee JB, Dewey J, Ballington JR (2004). Resveratrol, pterostilbene, and piceatannol in vaccinium berries.. J Agric Food Chem.

[3] Sanders TH, McMichael RW, Hendrix KW (2000). Occurrence of resveratrol in edible peanuts.. J Agric Food Chem.

[4] Burns J, Yokota T, Ashihara H, Lean ME, Crozier A (2002). Plant foods and herbal sources of resveratrol.. J Agric Food Chem.

[5] Signorelli P, Ghidoni R (2005). Resveratrol as an anticancer nutrient: molecular basis, open questions and promises.. J Nutr Biochem.

[6] Jang M, Cai L, Udeani GO, Slowing KV, Thomas CF (1997). Cancer chemopreventive activity of resveratrol, a natural product derived from grapes.. Science.

[7] Joe AK, Liu H, Suzui M, Vural ME, Xiao D (2002). Resveratrol induces growth inhibition, S-phase arrest, apoptosis, and changes in biomarker expression in several human cancer cell lines.. Clin Cancer Res.

[8] Harikumar KB, Kunnumakkara AB, Sethi G, Diagaradjane P, Anand P, et al. Resveratrol, a multitargeted agent, can enhance antitumor activity of gemcitabine in vitro and in orthotopic mouse model of human pancreatic cancer.. Int J Cancer.

[9] Pizarro JG, Verdaguer E, Ancrenaz V, Junyent F, Sureda F, et al. Resveratrol inhibits proliferation and promotes apoptosis of neuroblastoma cells: role of sirtuin 1.. Neurochem Res.

[10] Boissy P, Andersen TL, Abdallah BM, Kassem M, Plesner T (2005). Resveratrol inhibits myeloma cell growth, prevents osteoclast formation, and promotes osteoblast differentiation.. Cancer Res.

[11] Dai Z, Li Y, Quarles LD, Song T, Pan W (2007). Resveratrol enhances proliferation and osteoblastic differentiation in human mesenchymal stem cells via ER-dependent ERK1/2 activation.. Phytomedicine.

[12] Pandey PR, Okuda H, Watabe M, Pai SK, Liu W, et al..

[13] Yang YP, Chang YL, Huang PI, Chiou GY, Tseng LM, et al..

[14] Lu R, Serrero G (1999). Resveratrol, a natural product derived from grape, exhibits antiestrogenic activity and inhibits the growth of human breast cancer cells.. J Cell Physiol.

[15] Hsieh TC, Wu JM (1999). Differential effects on growth, cell cycle arrest, and induction of apoptosis by resveratrol in human prostate cancer cell lines.. Exp Cell Res.

[16] Benitez DA, Pozo-Guisado E, Alvarez-Barrientos A, Fernandez-Salguero PM, Castellon EA (2007). Mechanisms involved in resveratrol-induced apoptosis and cell cycle arrest in prostate cancer-derived cell lines.. J Androl.

[17] Wang TT, Hudson TS, Wang TC, Remsberg CM, Davies NM (2008). Differential effects of resveratrol on androgen-responsive LNCaP human prostate cancer cells in vitro and in vivo.. Carcinogenesis.

[18] Lee SK, Zhang W, Sanderson BJ (2008). Selective growth inhibition of human leukemia and human lymphoblastoid cells by resveratrol via cell cycle arrest and apoptosis induction.. J Agric Food Chem.

[19] Sexton E, Van Themsche C, LeBlanc K, Parent S, Lemoine P (2006). Resveratrol interferes with AKT activity and triggers apoptosis in human uterine cancer cells.. Mol Cancer.

[20] Hope C, Planutis K, Planutiene M, Moyer MP, Johal KS (2008). Low concentrations of resveratrol inhibit Wnt signal throughput in colon-derived cells: implications for colon cancer prevention.. Mol Nutr Food Res.

[21] Kimura Y, Okuda H (2001). Resveratrol isolated from Polygonum cuspidatum root prevents tumor growth and metastasis to lung and tumor-induced neovascularization in Lewis lung carcinoma-bearing mice.. J Nutr.

[22] Carbo N, Costelli P, Baccino FM, Lopez-Soriano FJ, Argiles JM (1999). Resveratrol, a natural product present in wine, decreases tumour growth in a rat tumour model.. Biochem Biophys Res Commun.

[23] Tessitore L, Davit A, Sarotto I, Caderni G (2000). Resveratrol depresses the growth of colorectal aberrant crypt foci by affecting bax and p21(CIP) expression.. Carcinogenesis.

[24] Sgambato A, Ardito R, Faraglia B, Boninsegna A, Wolf FI (2001). Resveratrol, a natural phenolic compound, inhibits cell proliferation and prevents oxidative DNA damage.. Mutat Res.

[25] Muller C, Ullmann K, Wilkens A, Winterhalter P, Toyokuni S (2009). Potent antioxidative activity of Vineatrol30 grapevine-shoot extract.. Biosci Biotechnol Biochem.

[26] Miura T, Muraoka S, Ikeda N, Watanabe M, Fujimoto Y (2000). Antioxidative and prooxidative action of stilbene derivatives.. Pharmacol Toxicol.

[27] Howitz KT, Bitterman KJ, Cohen HY, Lamming DW, Lavu S (2003). Small molecule activators of sirtuins extend Saccharomyces cerevisiae lifespan.. Nature.

[28] Gruber J, Tang SY, Halliwell B (2007). Evidence for a trade-off between survival and fitness caused by resveratrol treatment of Caenorhabditis elegans.. Ann N Y Acad Sci.

[29] Bauer JH, Goupil S, Garber GB, Helfand SL (2004). An accelerated assay for the identification of lifespan-extending interventions in Drosophila melanogaster.. Proc Natl Acad Sci U S A.

[30] Wood JG, Rogina B, Lavu S, Howitz K, Helfand SL (2004). Sirtuin activators mimic caloric restriction and delay ageing in metazoans.. Nature.

[31] Valenzano DR, Terzibasi E, Genade T, Cattaneo A, Domenici L (2006). Resveratrol prolongs lifespan and retards the onset of age-related markers in a short-lived vertebrate.. Curr Biol.

[32] Araki T, Sasaki Y, Milbrandt J (2004). Increased nuclear NAD biosynthesis and SIRT1 activation prevent axonal degeneration.. Science.

[33] Parker JA, Arango M, Abderrahmane S, Lambert E, Tourette C (2005). Resveratrol rescues mutant polyglutamine cytotoxicity in nematode and mammalian neurons.. Nat Genet.

[34] Wang Q, Yu S, Simonyi A, Rottinghaus G, Sun GY (2004). Resveratrol protects against neurotoxicity induced by kainic acid.. Neurochem Res.

[35] Wang Q, Xu J, Rottinghaus GE, Simonyi A, Lubahn D (2002). Resveratrol protects against global cerebral ischemic injury in gerbils.. Brain Res.

[36] Han YS, Zheng WH, Bastianetto S, Chabot JG, Quirion R (2004). Neuroprotective effects of resveratrol against beta-amyloid-induced neurotoxicity in rat hippocampal neurons: involvement of protein kinase C. Br J Pharmacol.

[37] Della-Morte D, Dave KR, DeFazio RA, Bao YC, Raval AP (2009). Resveratrol pretreatment protects rat brain from cerebral ischemic damage via a sirtuin 1-uncoupling protein 2 pathway.. Neuroscience.

[38] Bastianetto S, Zheng WH, Quirion R (2000). Neuroprotective abilities of resveratrol and other red wine constituents against nitric oxide-related toxicity in cultured hippocampal neurons.. Br J Pharmacol.

[39] Jin F, Wu Q, Lu YF, Gong QH, Shi JS (2008). Neuroprotective effect of resveratrol on 6-OHDA-induced Parkinson's disease in rats.. Eur J Pharmacol.

[40] Auger C, Teissedre PL, Gerain P, Lequeux N, Bornet A (2005). Dietary wine phenolics catechin, quercetin, and resveratrol efficiently protect hypercholesterolemic hamsters against aortic fatty streak accumulation.. J Agric Food Chem.

[41] Bradamante S, Barenghi L, Villa A (2004). Cardiovascular protective effects of resveratrol.. Cardiovasc Drug Rev.

[42] Wang Z, Zou J, Cao K, Hsieh TC, Huang Y (2005). Dealcoholized red wine containing known amounts of resveratrol suppresses atherosclerosis in hypercholesterolemic rabbits without affecting plasma lipid levels.. Int J Mol Med.

[43] Chen CK, Pace-Asciak CR (1996). Vasorelaxing activity of resveratrol and quercetin in isolated rat aorta.. Gen Pharmacol.

[44] Bertelli AA, Giovannini L, Bernini W, Migliori M, Fregoni M (1996). Antiplatelet activity of cis-resveratrol.. Drugs Exp Clin Res.

[45] Baur JA, Pearson KJ, Price NL, Jamieson HA, Lerin C (2006). Resveratrol improves health and survival of mice on a high-calorie diet.. Nature.

[46] Backesjo CM, Li Y, Lindgren U, Haldosen LA (2006). Activation of Sirt1 decreases adipocyte formation during osteoblast differentiation of mesenchymal stem cells.. J Bone Miner Res.

[47] Zhou H, Shang L, Li X, Zhang X, Gao G (2009). Resveratrol augments the canonical Wnt signaling pathway in promoting osteoblastic differentiation of multipotent mesenchymal cells.. Exp Cell Res.

[48] Rayalam S, Della-Fera MA, Baile CA Synergism between resveratrol, other phytochemicals: Implications for obesity, Mol Nutr Food Res 55: osteoporosis (1177–1185).

[49] Li Y, Danmark S, Edlund U, Finne-Wistrand A, He X, et al. Resveratrol-conjugated poly-epsilon-caprolactone facilitates in vitro mineralization and in vivo bone regeneration.. Acta Biomater.

[50] Rayalam S, Yang JY, Ambati S, Della-Fera MA, Baile CA (2008). Resveratrol induces apoptosis and inhibits adipogenesis in 3T3-L1 adipocytes.. Phytother Res.

[51] Yang JY, Della-Fera MA, Rayalam S, Ambati S, Hartzell DL (2008). Enhanced inhibition of adipogenesis and induction of apoptosis in 3T3-L1 adipocytes with combinations of resveratrol and quercetin.. Life Sci.

[52] Fischer-Posovszky P, Kukulus V, Tews D, Unterkircher T, Debatin KM, et al. Resveratrol regulates human adipocyte number and function in a Sirt1-dependent manner.. Am J Clin Nutr.

[53] Dong Z (2003). Molecular mechanism of the chemopreventive effect of resveratrol.. Mutat Res.

[54] Szende B, Tyihak E, Kiraly-Veghely Z (2000). Dose-dependent effect of resveratrol on proliferation and apoptosis in endothelial and tumor cell cultures.. Exp Mol Med.

[55] Clement MV, Hirpara JL, Chawdhury SH, Pervaiz S (1998). Chemopreventive agent resveratrol, a natural product derived from grapes, triggers CD95 signaling-dependent apoptosis in human tumor cells.. Blood.

[56] Ragione FD, Cucciolla V, Borriello A, Pietra VD, Racioppi L (1998). Resveratrol arrests the cell division cycle at S/G2 phase transition.. Biochem Biophys Res Commun.

[57] Shankar S, Nall D, Tang SN, Meeker D, Passarini J Resveratrol inhibits pancreatic cancer stem cell characteristics in human and KrasG12D transgenic mice by inhibiting pluripotency maintaining factors and epithelial-mesenchymal transition.. PLoS One.

[58] Filippi-Chiela EC, Villodre ES, Zamin LL, Lenz G Autophagy interplay with apoptosis, cell cycle regulation in the growth inhibiting effect of resveratrol in glioma cellsPLoSOne6:e2 (0849).

[59] Kaeberlein M, McDonagh T, Heltweg B, Hixon J, Westman EA (2005). Substrate-specific activation of sirtuins by resveratrol.. J Biol Chem.

[60] Borra MT, Smith BC, Denu JM (2005). Mechanism of human SIRT1 activation by resveratrol.. J Biol Chem.

[61] Walle T, Hsieh F, DeLegge MH, Oatis JE, Walle UK (2004). High absorption but very low bioavailability of oral resveratrol in humans.. Drug Metab Dispos.

[62] Nakagawa H, Kiyozuka Y, Uemura Y, Senzaki H, Shikata N (2001). Resveratrol inhibits human breast cancer cell growth and may mitigate the effect of linoleic acid, a potent breast cancer cell stimulator.. J Cancer Res Clin Oncol.

[63] Levenson AS, Gehm BD, Pearce ST, Horiguchi J, Simons LA (2003). Resveratrol acts as an estrogen receptor (ER) agonist in breast cancer cells stably transfected with ER alpha.. Int J Cancer.

[64] In K, Park J, Park H (2006). Resveratrol at high doses acts as an apoptotic inducer in endothelial cells.. Cancer Res Treat.

[65] Li Y, Liu J, Liu X, Xing K, Wang Y (2006). Resveratrol-induced cell inhibition of growth and apoptosis in MCF7 human breast cancer cells are associated with modulation of phosphorylated Akt and caspase-9.. Appl Biochem Biotechnol.

[66] Vyas S, Asmerom Y, De Leon DD (2006). Insulin-like growth factor II mediates resveratrol stimulatory effect on cathepsin D in breast cancer cells.. Growth Factors.

[67] Azios NG, Dharmawardhane SF (2005). Resveratrol and estradiol exert disparate effects on cell migration, cell surface actin structures, and focal adhesion assembly in MDA-MB-231 human breast cancer cells.. Neoplasia.

[68] J G, Cq W, Hh F, Hy D, Xl X (2006). Effects of resveratrol on endothelial progenitor cells and their contributions to reendothelialization in intima-injured rats.. J Cardiovasc Pharmacol.

[69] Fukui M, Yamabe N, Zhu BT Resveratrol attenuates the anticancer efficacy of paclitaxel in human breast cancer cells in vitro, in vivoEurJCancer46: (1882–1891).

[70] Aggarwal BB, Bhardwaj A, Aggarwal RS, Seeram NP, Shishodia S (2004). Role of resveratrol in prevention and therapy of cancer: preclinical and clinical studies.. Anticancer Res.

[71] Boocock DJ, Faust GE, Patel KR, Schinas AM, Brown VA (2007). Phase I dose escalation pharmacokinetic study in healthy volunteers of resveratrol, a potential cancer chemopreventive agent.. Cancer Epidemiol Biomarkers Prev.

[72] Chen T, Shen L, Yu J, Wan H, Guo A, et al..

[73] Song LH, Pan W, Yu YH, Quarles LD, Zhou HH (2006). Resveratrol prevents CsA inhibition of proliferation and osteoblastic differentiation of mouse bone marrow-derived mesenchymal stem cells through an ER/NO/cGMP pathway.. Toxicol In Vitro.

[74] Dienstknecht T, Ehehalt K, Jenei-Lanzl Z, Zellner J, Muller M, et al. Resazurin dye as a reliable tool for determination of cell number and viability in mesenchymal stem cell culture.. Bull Exp Biol Med.

[75] Dimri GP, Lee X, Basile G, Acosta M, Scott G (1995). A biomarker that identifies senescent human cells in culture and in aging skin in vivo.. Proc Natl Acad Sci U S A.

[76] Gyrd-Hansen M, Meier P IAPs: from caspase inhibitors to modulators of NF-kappaB, inflammation and cancer.. Nat Rev Cancer.

[77] Bilezikian JP RL, Rodan GA (2002). Principles of Bone Biology: Academic, London.

[78] Weiss MJ, Henthorn PS, Lafferty MA, Slaughter C, Raducha M (1986). Isolation and characterization of a cDNA encoding a human liver/bone/kidney-type alkaline phosphatase.. Proc Natl Acad Sci U S A.

[79] Zhao Y, Ding S (2007). A high-throughput siRNA library screen identifies osteogenic suppressors in human mesenchymal stem cells.. Proc Natl Acad Sci U S A.

[80] Rosen ED, Hsu CH, Wang X, Sakai S, Freeman MW (2002). C/EBPalpha induces adipogenesis through PPARgamma: a unified pathway.. Genes Dev.

[81] Calabrese EJ (2008). Hormesis and medicine.. Br J Clin Pharmacol.

[82] Calabrese EJ, Blain RB The hormesis database: The occurrence of hormetic dose responses in the toxicological literature..

[83] Calabrese EJ, Blain R (2005). The occurrence of hormetic dose responses in the toxicological literature, the hormesis database: an overview.. Toxicol Appl Pharmacol.

[84] Qian SW, Li X, Zhang YY, Huang HY, Liu Y, et al. Characterization of adipocyte differentiation from human mesenchymal stem cells in bone marrow.. BMC Dev Biol.

