# Supplementary figures and images for: Resveratrol Exerts Dosage and Duration Dependent Effect on Human Mesenchymal Stem Cell Development
Source: PLoS One. 2012 May 16;7(5):e37162. doi: 10.1371/journal.pone.0037162 (PMC3353901; doi:10.1371/journal.pone.0037162)

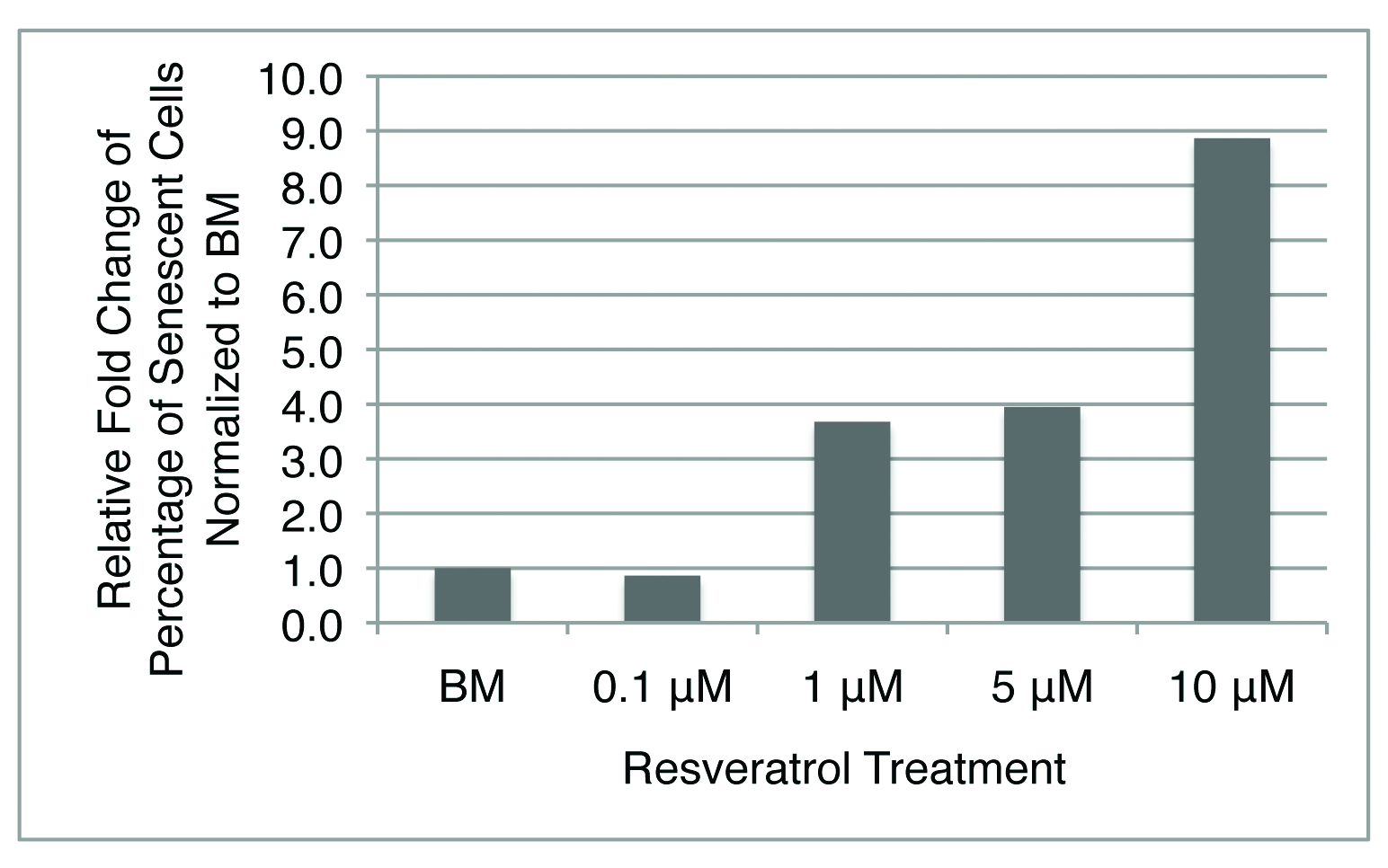

Supplement: Figure S1 — Resveratrol exerts dosage dependent anti- vs. pro-senescence effect on hMSCs. Percentages of senescent cells vs. total cells were determined based on images taken on X-gal stained cells pretreated with resveratrol or BM for 30 days followed by 5 more days of treatment after equal density plating. At least 283 total cells from each treatment condition were counted. Column represents the relative amount of cells undergoing senescence in each treatment group normalized to the value of the BM treated cells. Data was obtained from one experiment. (TIF) [file pone.0037162.s001.tif]

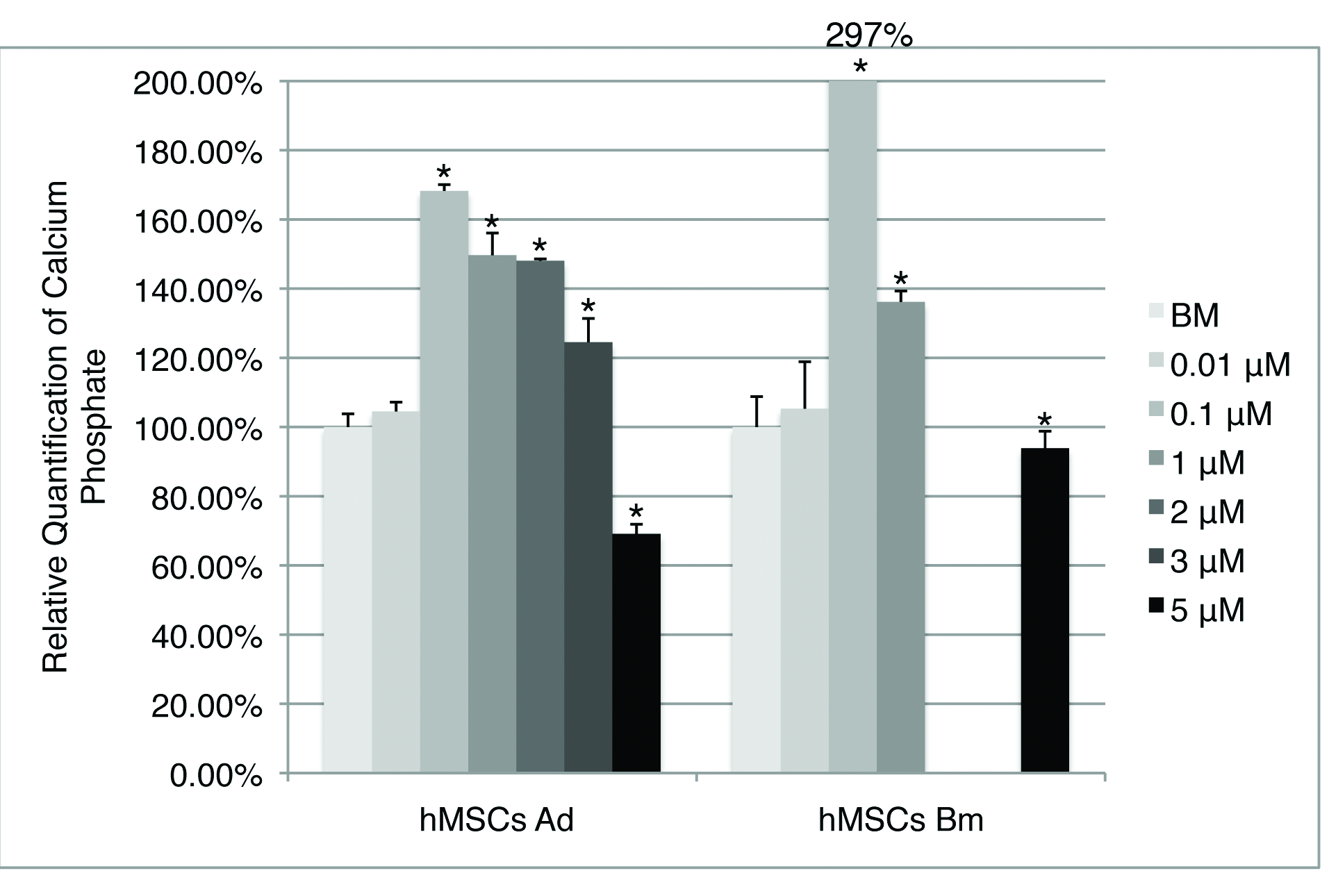

Supplement: Figure S2 — Resveratrol exerts dosage dependent effect on osteogenic differentiation of hMSCs derived from both the adipose tissue (hMSCs-Ad) and bone marrow (hMSCs-Bm). Cells were exposed to resveratrol/BM and OIM concurrently for 14 days before calcium phosphate assay. In hMSCs-Ad, resveratrol reached maximum enhancing effect at 0.1 µM, which gradually decreased as its concentration increased. Similar effect was also observed in hMSCs-Bm (concentrations at 2 and 3 µM were not examined). Data shown are the mean values of triplicates. Error bars represent standard deviation. *: p<0.05 vs. BM. (TIF) [file pone.0037162.s002.tif]
