# Supplementary material for: Resveratrol Exerts Dosage and Duration Dependent Effect on Human Mesenchymal Stem Cell Development
Source: PLoS One. 2012 May 16;7(5):e37162. doi: 10.1371/journal.pone.0037162 (PMC3353901; doi:10.1371/journal.pone.0037162)
Supplement: Table S1 — Resveratrol induces S-phase arrest in hMSCs in a dosage dependent manner. Flow cytometry was performed to analyze cell cycle distributions on cells pre-exposed to resveratrol or BM for 0 (0D-PT) or 30 (30D-PT) days, followed by 6 (0D-PT) or 4 (30D-PT) more days of treatment respectively after equal density plating, and cells cultured in regular hMSCs media (CM) for 30 days prior to 4 days of resveratrol or BM treatment following equal density plating (30D-CM). The percentage of cells in S, G2/M or (S+G2/M) phases in each treatment group was normalized to the value of the BM treated cells. Data presented are the mean value from 2 or 3 independent experiments for each experimental set, except for the 42D-PT set (cells pretreated with resveratrol or BM for 42 days were plated at equal density, followed by 4 more days of treatment before analysis), which was derived from a single experiment. All duplicate experiments had similar outcomes. (DOC) [file pone.0037162.s003.doc]

Supplement Table I:

| AVG-S phase | BM | 0.1uM | 1uM | 5uM | 10uM |
| --- | --- | --- | --- | --- | --- |
| 0D-PT | 4.52 | 5.05 | 5.27 | 6.04 | 8.39 |
| **%** | **100.0%** | **111.9%** | **116.6%** | **133.7%** | **185.7%** |
| 30D-PT | 6.35 | 7.23 | 7.65 | 14.66 | 18.38 |
| **%** | **100.0%** | **113.9%** | **120.6%** | **231.0%** | **289.6%** |
| 30D-CM | 4.01 | 4.29 | 4.26 | 9.04 | 12.64 |
| **%** | **100.0%** | **107.0%** | **106.2%** | **225.7%** | **315.6%** |
| 42D-PT | 2.57 | 3.06 | 2.78 | 5 | - |
| **%** | **100.0%** | **119.1%** | **108.2%** | **194.6%** | **-** |
|  |  |  |  |  |  |
| AVG-G2/M phase | BM | 0.1uM | 1uM | 5uM | 10uM |
| 0D-PT | 4.46 | 4.25 | 4.27 | 4.38 | 5.91 |
| **%** | **100.0%** | **95.1%** | **95.7%** | **98.1%** | **132.5%** |
| 30D-PT | 7.16 | 7.41 | 6.95 | 8.45 | 8.17 |
| **%** | **100.0%** | **103.5%** | **97.1%** | **118.0%** | **114.1%** |
| 30D-CM | 4.62 | 4.43 | 5.04 | 6.80 | 6.61 |
| **%** | **100.0%** | **95.9%** | **109.1%** | **147.2%** | **143.1%** |
| 42D-PT | 1.95 | 1.83 | 2.15 | 3.90 | - |
| **%** | **100.0%** | **93.8%** | **110.3%** | **200.0%** | **-** |
|  |  |  |  |  |  |
| AVG (S+G2/M) phase | BM | 0.1uM | 1uM | 5uM | 10uM |
| 0D-PT | 8.98 | 9.30 | 9.54 | 10.42 | 14.30 |
| **%** | **100.0%** | **103.6%** | **106.2%** | **116.0%** | **159.2%** |
| 30D-PT | 13.50 | 14.64 | 14.60 | 23.10 | 26.54 |
| **%** | **100.0%** | **108.4%** | **108.1%** | **171.1%** | **196.6%** |
| 30D-CM | 8.62 | 8.71 | 9.29 | 15.84 | 19.25 |
| **%** | **100.0%** | **101.0%** | **107.8%** | **183.7%** | **223.3%** |
| 42D-PT | 4.52 | 4.89 | 4.93 | 8.90 | - |
| **%** | **100.0%** | **108.2%** | **109.1%** | **183.7%** | **196.9%** |
