# Supplementary material for: Resveratrol Exerts Dosage and Duration Dependent Effect on Human Mesenchymal Stem Cell Development
Source: PLoS One. 2012 May 16;7(5):e37162. doi: 10.1371/journal.pone.0037162 (PMC3353901; doi:10.1371/journal.pone.0037162)
Supplement: Table S2 — Primer sequences and PCR conditions. This table enlists the primer sequences and PCR conditions used for gene expression examination by RT-PCR. (DOCX) [file pone.0037162.s004.docx]

**Supplement Table II**

| **Gene name (hs.)** | **Forward primer 5'-3'** | **Reverse Primer 5'-3'** | **Product size (bps)** | **PCR condition** |
| --- | --- | --- | --- | --- |
| HSP90-beta | TACTTGGTGGCAGAGAAAGT | CTCATCTGAACCCACATCTT | 363 | 90C 30'', 60C 1', 72C 1' |
| PPARgamma2 | AAG CCC TTC ACT ACT GTT GA | ACC TGA TGG CAT TAT GAG AC | 444 | 90C 30'', 60C 1', 72C 1' |
| CEBP alpha | CCT AAG GTT GTT CCC CTA GT | GAG AGT CTC ATT TTG GCA AG | 547 | 90C 30'', 58C 1', 72C 1' |
| Osteocalcin | CTACCTGTATCAATGGCTGG | CAGATTCCTCTTCTGGAGTTTA | 310 | 90C 30'', 53C 1', 72C 1' |
| ALPL | TGG AGC TTC AGA AGC TCA ACA CCA | ATC TCG TTG TCT GAG TAC CAG TCC | 450 | 90C 30'', 60C 1', 72C 1' |
| BIRC4 | ACCCGAGGAACCCTGCCATGT | TGCAGGCGCCTTAGCTGCTC | 874 | 90C 30'', 63C 1', 72C 1' |
| BIRC5 | AGCATTCGTCCGGTTGCGCT | TTACCAGCAGCACCCGCTGC | 403 | 90C 30'', 63C 1', 72C 1' |
| SIRT1 | GGCGGCTGGGGAAGGAGACAAT | CATCAGCTGGGCACCTAGGACA | 917 | 90C 30'', 63C 1', 72C 1' |
| SIRT2 | ATGTCTGCTGAGTTGTAGTTC | ATGGTACTTCTCTAGGTTGTCATAG | 503 | 90C 30'', 55C 1', 72C 1' |
| CDK2 | TGACTCGCCGGGCCCTATTCC | CCCAAGGCCAAGCCTGGTCA | 381 | 90C 30'', 63C 1', 72C 1' |
| P21 | GCGCCATGTCAGAACCGGCT | GCCGCCGTTTTCGACCCTGA | 435 | 90C 30'', 63C 1', 72C 1' |
